# New Insights toward Colorectal Cancer Chemotherapy Using Natural Bioactive Compounds

**DOI:** 10.3389/fphar.2017.00109

**Published:** 2017-03-14

**Authors:** Saúl Redondo-Blanco, Javier Fernández, Ignacio Gutiérrez-del-Río, Claudio J. Villar, Felipe Lombó

**Affiliations:** Departamento de Biología Funcional, Área de Microbiología, Facultad de Medicina, Instituto Universitario de Oncología del Principado de Asturias (IUOPA), Universidad de OviedoOviedo, Spain

**Keywords:** CRC, nutraceutical, chemotherapy, radiotherapy, combination therapy, apoptosis

## Abstract

Combination therapy consists in the simultaneous administration of a conventional chemotherapy drug (or sometimes, a radiotherapy protocol) together with one or more natural bioactives (usually from plant or fungal origin) of small molecular weight. This combination of anticancer drugs may be applied to cell cultures of tumor cells, or to an animal model for a cancer type (or its xenograft), or to a clinical trial in patients. In this review, we summarize current knowledge describing diverse synergistic effects on colorectal cancer cell cultures, animal models, and clinical trials of various natural bioactives (stilbenes, flavonoids, terpenes, curcumin, and other structural families), which may be important with respect to diminish final doses of the chemotherapy drug, although maintaining its biological effect. This is important as these approaches may help reduce side effects in patients under conventional chemotherapy. Also, these molecules may exerts their synergistic effects via different cell cycle pathways, including different ones to those responsible of resistance phenotypes: transcription factors, membrane receptors, adhesion and structural molecules, cell cycle regulatory components, and apoptosis pathways.

## Introduction

CRC is the third most common cancer in men (after lung and prostate cancers) and the second in women (after breast cancer) worldwide, with a prevalence of 10.0 and 9.2%, respectively (Merrill and Anderson, 2011; Bray et al., 2013; Ferlay et al., 2015). CRC is also one of the leading death causes and, despite the improvement in our knowledge in this disease achieved in recent years, current treatments are not enough to control metastatic forms of CRC (Santandreu et al., 2011). Surgery is the main procedure in patients with potentially curable CRC, but neoadjuvant chemotherapy and/or radiotherapy is sometimes given before or after surgery depending on disease stage. However, these treatment regimens are not enough to control CRC, since 30% of patients with stage I–III and up to 65% of patients with stage IV will develop recurrent disease (van der Stok et al., 2016), highlighting the urgency of finding new and more effective treatment schemes.

The potential of nutraceutical natural compounds such as flavonoids, anthocyanidins, carotenoids, or terpenoids for cancer prevention has been widely investigated, and there are many evidences supporting that moderate consumption of fruits and vegetables is correlated with decreased risk of CRC (Fernández et al., 2016). Some members of these families of compounds have the ability to modulate signaling pathways as well as to regulate the expression of genes involved in cell cycle regulation, differentiation, and apoptosis (Pan et al., 2011). Besides being useful in prevention, some of these molecules could be also helpful for the treatment of CRC, especially in combination with other drugs.

Combination therapy allows targeting simultaneously different pathways involved in cancer, taking advantage of different mechanisms of action in order to reduce the development of tumor drug resistance (Housman et al., 2014). In the case of CRC, diverse cell cycle alterations are involved in its establishment and development, as in the case of chromosomal instability versions (CIN; around two-thirds of cases), the DNA mismatch repair phenotype (around 15% of CRC cases) and other less frequent CRC versions as abnormal DNA methylation, colon inflammation status, and microRNA triggering effects (Colussi et al., 2013). In CIN CRC phenotypes, for example, diverse signaling pathways become affected, as those involving APC, β-catenin, Tcf, and WNT proteins (Morin et al., 1997; Sparks et al., 1998). Several studies published in recent years have shown that cancer treatment through combinatorial approach is much more effective than the use of drugs individually (Singh et al., 2013). Also, chemosensitization by means of phytochemicals, based on the use of a natural compound to increase the activity of a drug through modulation of its resistance pathways, is one of the strategies proposed to overcome chemoresistance, one of the main challenges in CRC treatment (Amiri et al., 2013).

Combinations of two drugs onto a biological system may produce improved (synergistic), reduced (antagonistic), or identical (additive) effects compared to their effects when acting separately. Since combinatorial approach to cancer treatment with natural compounds is a promising way to avoid resistances (by affecting more than one target) and to enhance the potency of chemotherapy (through chemosensitization; Majumdar et al., 2009; Gupta et al., 2011), it is necessary for researchers to mathematically assess the nature of these interactions between molecules. This is often made by using the Chou-Talay combination index (CI), based on the median-effect equation: *CI* = *a/A* + *b/B* (Chou and Talalay, 1984). *A* and *B* are, respectively, the doses of drug *A* (alone) and *B* (alone) needed to produce a specified effect while *a* and *b* is the dose in combination that produces the same effect. CI shows an additive interaction between two drugs when it is equal to 1, synergism when CI < 1, and antagonism when CI > 1 (Tallarida, 2002).

## Stilbenes

**Figure 1:**
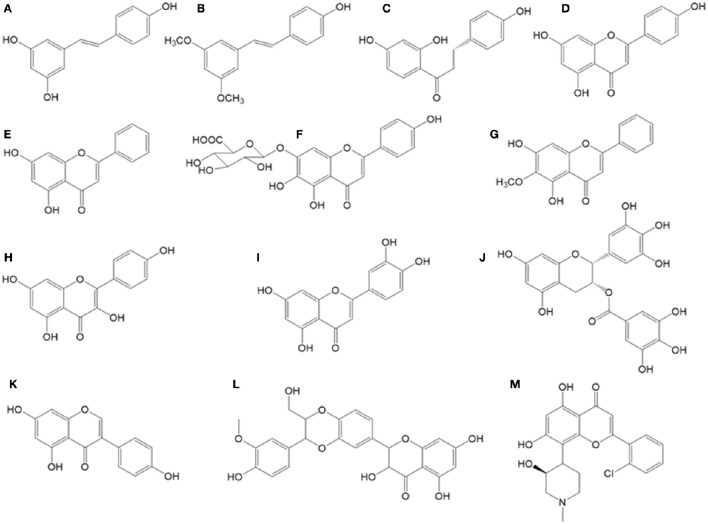
**Chemical structures of bioactive stilbenes and flavonoids described in the text. (A)** Resveratrol, **(B)** pterostilbene, **(C)** isoliquiritigenin, **(D)** apigenin, **(E)** chrysin, **(F)** quercetin, **(G)** oroxylin, **(H)** kaempferol, **(I)** genistein, **(J)** flavopiridol, **(K)** silibinin, **(L)** scutellarin, **(M)** EGCG.

Resveratrol (Figure 1A) is a stilbene found in more than 70 plant species, including edible plants such as grapes, raspberries, blueberries, or peanuts, and the Japanese knotweed (*Polygonum cuspidatum*), which contains the highest naturally occurring levels of this molecule (Burns et al., 2002). Resveratrol is a phytoalexin, a natural inhibitor of cell proliferation, synthesized by plants in response to environmental stress and pathogenic invasion (Singh et al., 2013).

Since the publication in 1997 of the chemopreventive activity of topically applied resveratrol in a mouse model of skin cancer (Jang et al., 1997), this natural compound has been extensively studied for the prevention and also treatment of many diseases, including CRC. The promising *in vitro* results of these studies have made resveratrol one of the natural compounds that have attracted more attention in recent times, even among mainstream media. For example, resveratrol can interfere with some hallmarks of cancer, protecting against both tumor initiation and cancer progression by interfering with cytochrome P_450_ isoenzymes, inhibiting cyclooxygenase (COX) enzymes and decreasing DNA binding activity of NF-kB, which is usually upregulated in cancer. Resveratrol can also mimic the effects of caloric restriction and protect against metabolic disease, through activation of the SIRT1 histone deacetylase and AMPK (Gescher et al., 2013; Carter et al., 2014).

Beyond its potential usefulness in cancer chemoprevention or even treatment, recent studies have shown that resveratrol can exert synergistic activities used in combination with other chemotherapeutic agents (Table 1). This would allow to establish a new and more effective treatment with fewer side effects (Singh et al., 2013). An overview of the studies published to date analyzing combinations of resveratrol with antitumor drugs is reviewed below.

**Table 1:** **Summary of main ***in vitro*** and ***in vivo*** synergistic effects of combinations of stilbenes and chemotherapeutic compounds against CRC**.

**References**	**Tested molecule**	**In combination with**	**Experimental model**	**Main result**	**Proposed mechanism**
Ali and Braun, 2014	Resveratrol	Mitomycin C	CRC cell culture (primary cell lines from resected colorectal tumors)	Synergistic suppression of cell proliferation by resveratrol and Mitomycin C	Up-regulation of p21^WAF1/CIP1^
Amiri et al., 2013	Resveratrol	Etoposide	CRC cell culture (HCT-116)	Synergistic effect of resveratrol on etoposide	Up-regulation of *TP53* expression
Buhrmann et al., 2015	Resveratrol	5-Flourouracil	CRC cell culture (HCT-116, SW480) in a 3D-alginate microenviroment	Synergistic activity between resveratrol and 5-Fu decreasing viability and inducing apoptosis	Up-regulation of desmosomes, gap and tight junction adhesion molecules. Inhibition of EMT factors. Down-regulation of NF-kB activation
Kaminski et al., 2014	Resveratrol	Oxaliplatin	CRC cell culture (Caco-2)	Positive: CRC cells chemosensitization by resveratrol. Synergistic activity of resveratrol and oxaliplatin inhibiting CRC cell growth	Induction of cell death
Kumazaki et al., 2013	Resveratrol	5-Fluorouracil	CRC cell culture (DLD-1, SW480, COLO201)	Synergistic enhancement of growth inhibition and apoptosis	Up-regulation of miR-34a expression causing a down-regulation of *E2F3*
Majumdar et al., 2009	Resveratrol	Curcumin	CRC cell culture (HCT-116) and mouse xenograft CRC models	Synergism between curcumin and resveratrol inhibiting growth of CRC cells *in vitro* and *in vivo*	Attenuation of NF-κB activity. Inhibition of constitutive activation of EGFR
Mohapatra et al., 2011	Resveratrol	5-Fluorouracil	CRC cell culture (HCT-116)	Synergistic induction of apoptosis	Cell cycle arrest in S phase, enhanced DNA damage
Santandreu et al., 2011	Resveratrol	5-Fluorouracil	CRC cell culture (HT-29, SW620)	Positive: Resveratrol sensitize CCR cells to 5-Fluorouracil	Increase in oxidative stress, inactivation or down-regulation of redox-sensitive proteins
Yang S. et al., 2015	Resveratrol	Oxaliplatin	CRC cell culture (HCT-116, HT-29) and mouse xenograft CRC model	Synergistic effect of resveratrol and oxaliplatin in a *miR-34c* dependent manner	Up-regulation of *miR-34c*
Fulda and Debatin, 2004	Resveratrol	5-Fluorouracil	CRC cell culture (HCT-116) and other human cancer cell lines	Positive: Resveratrol sensitizes CRC cells for subsequent treatment with 5-Fu	Cell cycle arrest and apoptosis by downregulation of surviving, irrespective of p53 status
Hwang et al., 2007a	Resveratrol	Etoposide	CRC cell culture (HT-29)	Positive: Resveratrol chemosensitizes CRC cells for subsequent treatment with etoposide	inhibition of cell growth, increase of ROS generation, activation of AMPK, induction of apoptosis
Tolba and Abdel-Rahman, 2015	Pterostilbene	5-Fluorouracil	CRC cell culture (HCT-116, Caco-2)	Synergistic effect of pterostilbene on cytotoxic effects of 5-FU	Supression of Akt and ERK phosphorylation. Increase of FOXO-1 and p27kip1 levels

Combinations of resveratrol and quercetin have been shown to synergistically induce apoptosis in the MOLT-4 leukemia cell line (Mertens-Talcott and Percival, 2005). Based on this study, the effects of different concentrations of a 1:1 combination of quercetin and resveratrol on the HT-29 CCR cell line were analyzed, focusing on its effect on Sp transcription factors, usually overexpressed in tumors. The combination of both compounds induced apoptosis in the HT-29 line, decreasing RNA and protein levels of survivin, Sp1, Sp3, and Sp4, in a pathway in which the microRNA-27a appears to be involved. It should be noted that in this study no synergistic effect can be detected between the two compounds since all the tests were carried out with combinations of both compounds and their effects alone were not analyzed (Del Follo-Martinez et al., 2013).

Using an azoxymethane-induced mouse model of colon carcinogenesis, recent studies showed that a combination of resveratrol and grape seed extract reduced the incidence of tumors as much as the nonsteroidal anti-inflammatory drug sulindac, without occurrence of toxicity. In addition, *in vitro* assays performed with isolated human colon cancer stem cells (CSCs) showed that this combination of compounds suppressed proliferation, sphere formation and nuclear translocation of β-catenin through the downregulation of c-Myc and cyclin D, downstream proteins of Wnt/β-catenin pathway (Reddivari et al., 2016).

In 2004, it was discovered that resveratrol sensitizes HCT116 CRC cells to 5-Fluorouracil (5-FU; Fulda and Debatin, 2004). The first experiments were conducted in SHEP neuroblastoma cells, finding that resveratrol induces apoptosis in cooperation with several antitumor drugs (VP16, doxorubicin, cytarabine, actinomycin D, taxol, or methotrexate). Interestingly, pretreatment with resveratrol prior to exposure of these antitumor drug was more effective than concurrent or subsequent treatment. In order to test the role of p53 in the chemosensitizing effect of resveratrol, additional experiments were performed with wild-type p53 and p53-deficient HCT116 CRC cells. Pretreatment with 30 μM resveratrol for 24 h increased apoptosis induced by 5-FU (at 10, 30, and 100 μM during the next 24 h) on both cell lines. These results suggest that resveratrol can induce cell cycle arrest and apoptosis independently of p53 status (Fulda and Debatin, 2004). Nevertheless, other researchers have reported that p53 upregulation could play an important role on the synergistic effect between resveratrol and etoposide, a topoisomerase II inhibitor used as an antineoplastic drug (Amiri et al., 2013).

Resveratrol also sensitizes HT-29 and SW620 CRC cell lines to cytotoxic oxidative stress induced by 5-FU, by inhibiting their endogenous antioxidant capacity (Santandreu et al., 2011). Moderate resveratrol concentration (15 μM) in combination with very low 5-FU (0.5 μM) concentration causes a significant inhibition of cell proliferation, migration, and cell cycle arrest at S phase, leading to apoptosis in HCT-116 cells. The same study provides evidence suggesting that its mechanism of action may be related with the activation of the MAPK pathway through upregulation of p-JNK and p-p38, with no p-ERK changes (Mohapatra et al., 2011). Similar results were found in a study with etoposide resistant HT-29 cells, where resveratrol was able to chemosensitize HT-29 cells promoting cell cycle inhibition, reactive oxygen species (ROS) generation, AMPK activation, and apoptosis induction (Hwang et al., 2007a).

Inter-cellular junctions could play an important role in the synergism observed between resveratrol and 5-FU, a drug which can induce an increase of mesenchymal features and loss of epithelial ones in CRC cells; those related to cancer proliferation, motility, drug resistance and metastasis. Resveratrol chemosensitizes CRC cells to 5-FU through inhibition of EMT (epithelial–mesenchymal transition) factors (vimentin and SNAI2 proteins), up-regulation of intercellular junctions (desmosomes, gap and tight junctions, and adhesion molecules such as E-cadherin) and by down-regulation of NF-kB pathway (Table 1; Buhrmann et al., 2015).

A new mechanism based on miR-34a has been also described which may partially explain the synergistic inhibition of HCT116 growth induced by resveratrol and 5-FU. Here, resveratrol promoted suppression of PI3K/Akt and MAPK Erk1/2 signaling pathways and upregulation of miR-34a expression, which downregulates *E2F3* gene expression and its downstream target *Sirt1* gene (Kumazaki et al., 2013).

Other recent study shows that resveratrol chemosensitizes HT-29 and HCT-116 CRC cells to oxaliplatin through up-regulation of miR-34c, which in turn knocked down its target KITLG. This result was confirmed in xenograft mice, where the combination treatment with oxaliplatin and resveratrol was more effective inhibiting tumor growth than individual treatments (Yang S. et al., 2015). Also, resveratrol and oxaliplatin combinations synergistically inhibit cell growth of Caco-2 CRC cells via apoptosis and necrosis induction (Kaminski et al., 2014).

Mitomycin C is another drug that can be potentially enhanced by resveratrol. Unlike mitomycin C, resveratrol can induce p21^WAF1/CIP1^ overexpression regardless of p53 status, and a combined treatment of these two compounds has inhibited synergistically the proliferation of mitomycin C-resistant CRC cells (Ali and Braun, 2014).

Pterostilbene (Figure 1B), a structural analog to resveratrol, characterized by the presence of two methoxy groups instead of resveratrol hydroxyl groups, is able to enhance 5-FU treatment in CRC cells. This synergistic effect is stronger in Caco-2 cells, which express higher levels of ER-β (estrogen receptor beta) compared with HCT116 cells (Tolba and Abdel-Rahman, 2015).

Despite all these promising features of resveratrol as chemopreventive, chemotherapeutic and chemosensitizer agent, clinical trials and other *in vivo* evidences suggest that there may be limitations in clinical application, mainly due to its low systemic availability. Between 70 and 80% of orally consumed resveratrol is quickly absorbed via passive diffusion in the enterocytes. After that, conjugated resveratrol derivatives (glucuronides and sulfates) are rapidly formed, and only 2% of unmodified trans-resveratrol is found in the blood, reaching its maximum concentration between 30 and 60 min after ingestion (Carter et al., 2014). For example, a single 25 mg dose of resveratrol results in a 2 mM (490 ng/mL) serum peak for resveratrol and all of its metabolites, with only trace amounts of unmodified resveratrol (< 5 ng/mL; Walle et al., 2004). Furthermore, it has been observed that resveratrol absorption and pharmacokinetics are strongly influenced by food matrix (Rotches-Ribalta et al., 2012) and by its metabolism by gastrointestinal microbiota (Bode et al., 2013). Also, resveratrol dosage in patients and volunteers over 1 g per day has shown gastrointestinal adverse effects such as diarrhea, flatulence, nausea, and abdominal pain (Brown et al., 2010). Anyway, resveratrol and its metabolites have been identified in normal and tumor colorectal human tissue samples, in higher concentrations than those found in blood samples after intake doses of 0.5–1 g/day. Thus, colorectum is a suitable target tissue for chemoprevention and combination therapy by oral resveratrol, as observed concentrations in this tissue are able to produce pharmacological effects (Patel et al., 2010).

## Flavonoids

Flavonoids are one of the most numerous and widely distributed family of bioactive compounds in plants. These polyphenolic secondary metabolites are essential for plants morphology and physiology. Flavonoids are involved in flowers, seeds, stems, and leaves pigmentation, as well as in its growth and reproduction (to attract pollinators), while at the same time they protect plants against microbial infections and ultraviolet radiation (Harborne and Williams, 2000). Chemically, flavonoids are characterized by showing a 15-C skeleton (structured as C6-C3-C6) with two phenyl aromatic rings (A and B) plus one heterocycle aromatic ring (ring C), all of them tailored with one or more hydroxyl groups (Manach et al., 2004). Flavonoids are further subdivided into several subgroups depending on the degree of substitution: chalcones (as isoliquiritigenin); flavanones (as naringenin), flavones (as apigenin and luteolin), flavonols (as quercetin and kaempferol), flavanols (as epigallocatechin), isoflavones (as genistein), and anthocyanins. Flavonoids are the largest group of diet polyphenols, with more than 4,000 representatives (Manach et al., 2004; Kumar and Pandey, 2007).

Although, flavonoids are not necessary nutrients for well-being in the short-term, there are several evidences that claim that a moderate intake has beneficial long-term health effects. These compounds, the same as stilbenes, are powerful antioxidants that prevent the appearance of tumors, cardiovascular diseases and osteoporosis, improve cognitive functions and diabetes; or have phytoestrogenic, anti-inflammatory, antibacterial, or antiviral actions, having therefore a strong impact on human health (Kumar and Pandey, 2013).

### Isoliquiritigenin

Isoliquiritigenin (Figure 1C), a chalcone originated from dried roots of several *Glycyrrhiza* species (licorice plants), exhibits antioxidant, estrogenic, and anti-tumor activities (Guo et al., 2008). It has shown synergistic effect in combination with cisplatin in a xenograft mice model for CRC using CT-26 mouse CRC cells. In this mice model, an oral dose of 1 mg/kg of this chalcone, plus an intraperitoneal cisplatin injection of 5 mg/kg were able to reduce 79% the tumor growth. Also, addition of isoliquiritigenin to this cisplatin treatment was able to reduce liver and kidney damage, as transaminases (AST, ALT), creatinine and blood urea nitrogen levels were kept at normal concentrations, in contrast with control cisplatin treatment. With respect to oxidative damage, this combination therapy with isoliquiritigenin reduced nitric oxide serum levels, lipid peroxidation and GSH levels, in contrast to cisplatin treatment alone (Table 2; Lee et al., 2008). This point is very interesting, as major hepatic damages caused by cisplatin are bound to increased oxidative damage due to depletion in GSH levels and an increase in malonaldehyde and membrane peroxidation. Cisplatin treatment is also associated to increased serum levels of transaminases and bilirubin, two important markers for hepatic damage, following histopathological changes as necrosis and hepatocytes degeneration with infiltration of inflammatory cells around portal vein (Caro and Cederbaum, 2004; Dasari and Tchounwou, 2014). Increased liver damage due to cisplatin has been observed in patients with higher expression levels of cytochrome P450-2E1 (Caro and Cederbaum, 2004). The reduction achieved in cisplatin doses, if transferred to *in vivo* experiments, would contribute to a potential reduction in side effects caused by this drug, which usually are associated to ototoxicity, gastrotoxicity, myelosuppression, hepatotoxicity (due to ROS causing a reduction in GSH levels and an increase in malonaldehyde), cardiotoxicity (due to depletion in cardiac myocytes of lactate dehydrogenase and creatine kinase, following membrane peroxidation in these cells), and nephrotoxicity (due to inhibition of carnitine synthesis and its reabsorption by the proximal tube; Dasari and Tchounwou, 2014) (**Figure 3**).

**Table 2:** **Summary of main ***in vitro*** and ***in vivo*** synergistic effects of combinations of flavonoids and chemotherapeutic compounds against CRC**.

**References**	**Tested molecule**	**In combination with**	**Experimental model**	**Main result**	**Proposed mechanism**
Horinaka et al., 2006	Apigenin	TRAIL	CRC cell culture (DLD-1)	Synergistic potentiation of TRAIL-induced apoptosis	Up-regulation of DR5
Shao et al., 2013	Apigenin	ABT-263 (Navitoclax)	CRC cell culture (HTC-116) and SCID mice bearing HTC-116 xenografts	Synergistic induction of apoptosis, antagonism effect on ABT-263-induced Mcl-1 up-regulation and greater tumour growth inhibition	Down-regulation of Mcl-1, inhibition of PI3K/AKT pathway and ERK phosphorylation
Yoshida et al., 2008	Kaempferol	TRAIL	CRC cell culture (SW480 and DLD-1)	Positive: Increase in apoptotic induction in a kaempferol-dose dependent manner	Up-regulation of DR5
Li et al., 2010	Chrysin	TNFα	CRC cell culture (HCT-116)	Positive: Increase in cell death	Inhibition of TNFα-induced NF-κβ activation
Khan et al., 2012	Chrysin	Cisplatin	Wistar rats	Positive: Prophylactic effect against colon toxicity	Reducing oxidative stress
Ding et al., 2012	Chrysin	TRAIL	CRC cell culture (HT-29)	Positive: Enhanced TRAIL-induced cell death	Suppression of c-FLIP and up-regulation of DR5
León et al., 2015	Chrysin	Vanadyl cation	CRC cell culture (HT-29)	Positive: Cell cycle arrest in G2/M phase	Decrease in GSH levels
Hwang et al., 2005	Genistein	5-FU	CRC cell culture (HT-29)	Synergistic effect on cell growth blocking	Over-expression of pro-apoptotic p53 and p21, down-regulation of Glut-1 and down-regulation of COX-2
Hu et al., 2014	Genistein	Cisplatin	CRC cell culture (HT-29)	Positive: Inhibited cell growth and induced apoptosis in an additive manner	Inhibition of TK
Park et al., 2001	Genistein	Dexamethasone	CRC cell culture (Colo320 HSR)	Synergistic effect on blocking cell cycle	Increase in p21 levels
Son et al., 2013	Genistein	Radiotherapy	BALB/c mice bearing CT26 xenografts	Positive: Less non-tumorigenic apoptotic cells and improved morphological changes in healthy intestinal tissue	Activation of antioxidant systems
Gruca et al., 2014	Genistein	Radiotherapy	CRC cell culture (HCT-116)	Synergistic effect on clonogenic survival	Enhanced EGFR inhibition and prolonged inhibition of AKT and ERK
Kumazaki et al., 2013; Wubetu et al., 2015	EGCG	5-FU	CRC cell culture (DLD-1, SW480 and COLO201)	Synergistic growth suppression	Regulation of ABC transporter-related genes
Saldanha et al., 2014	EGCG	Sodium butyrate	CRC cell culture (HT-29)	Synergistic induction of apoptosis	Down-regulation of survivin
Ohishi et al., 2002	EGCG	Sulindac	Azoxymethane colon cancer induction in rats	Synergistic induction of apoptosis	Enhanced inhibition of COX-2
Ambrosini et al., 2008	Flavopiridol	SN-38	HCT116 cell line	Synergistic effect on the apoptotic effects of SN-38	Down-regulation of Rad51 by p53 and Cdk9 inhibition.
Darpolor et al., 2011	Flavopiridol	Irinotecan	Mice xenograft (HCT116)	Improves tumor response	Reduces cytokine activity
Fornier et al., 2007	Flavopiridol	Docetaxel	Phase I trial	Partial responses and a complete response in one patient	Unknown
Guo et al., 2006	Flavopiridol	Docetaxel and 5-FU	Mice xenograft (HCT116)	Significant decrease in a tumor volume	Unknown
Motwani et al., 2001	Flavopiridol	SN-38	Mouse xenograft model	Flavopiridol enhances a reduction in tumors	The effect is produced by p21
Colombo et al., 2011	Silymarin	Doxorubicin and paclitaxel	LoVo cell line	Synergistic effect in LoVo cells and additive in LoVo/DX	Low expression of p-gp pump
León et al., 2015	Silymarin	Vanadium compounds	HT29 cell line	Improves cytotoxic effect	Inhibits topoisomerase IB activity and NF-κB
Tsai et al., 2015	Silibinin	Metformin	COLO205 cell line	Reduction cell viability more than 60%	Increase caspase 3 activation and AIF expression
Psahoulia et al., 2007	Quercetin	TRAIL	Caco-2, SW620 and HT29 cell lines	Sensitizes the cells to the treatment	Distribution of death factors in raft domains that are the initiators of apoptosis
Xavier et al., 2011	Quercetin	5-FU	Co115 and HCT15 cell lines	Enhances apoptosis more than 100 times	The effect is mediated by p53
Osman et al., 2015	Luteolin	Aspirin	Colorectal cancer in rats	Highly significant reduction in polyps number and size	Enhance inhibition the inflammatory response
Chan et al., 2009	Scutellarin	5-FU	HCT116 cell line	A significant increase in apoptosis levels	p53-regulated caspase-6 activation mechanism
Lee et al., 2008	Isoliquiritigenin	Cisplatin	Mice xenograft (CT26)	Reduce 79% tumor growth and reduces adverse effects	Mechanism in combination is unknown
Ha et al., 2012	Oroxylin	5-FU	HT29 cell line and mice xenografts (HT29)	Reduce 66% tumor growth and shows synergistic effects in HT29 cell line	Inhibition of COX-2 gene expression
Cheah et al., 2014	Procyanidins	5-FU	Caco-2 cell line	Increase cytotoxicity	Unknown

### Apigenin

Apigenin (Figure 1D) is one of the most widely distributed flavones in fruits and vegetables, such as parsley, Chinese cabbage, bell pepper, garlic, celery, and guava (Manach et al., 2004). It is a chemopreventive agent that has been shown to present strong cytostatic and anti-angiogenic effects *in vitro* (Hirano et al., 1989; Engelmann et al., 2002). *In vitro*, apigenin induces growth inhibition, cell cycle arrest, and apoptosis in CRC cells (Zhong et al., 2010; Lee Y. et al., 2014; Yang L. et al., 2015). Moreover, apigenin is a strong inhibitor of ABC transporters (ATP-Binding Cassette), which are responsible for the increase in the efflux of chemotherapeutic drugs in the lumen (apical) face of colonocytes, thereby significantly reducing its bioavailability and leading to its active detoxification (Katayama et al., 2007). Finally, apigenin is also responsible for NAG-1 [Nonsteroidal Anti-inflammatory Drug (NSAID) Activated Gene-1] overexpression in CRC cells, a member of the TGF-B (Transforming Growth Factor-B) superfamily which shows pro-apoptotic and antitumor activities (Yang et al., 2014). In fact, apigenin increases in a dose-dependent way in CRC cells, both *in vivo* and *in vitro*, NAG-1 and p53 expression, reducing intestinal tumor load and number (Zhong et al., 2010). Therefore, apigenin has a promising application as a safe antitumor agent. However, it has a modest antitumor activity against cancer cells when used alone, so new strategies are needed in order to enhance its effectiveness, as those based on combination therapy of this flavonoid with CRC drugs.

CD26 is a multifunctional cell-surface protein that is involved in the suppression of pathways responsible for tumor growth and metastasis. In fact, CD26 is down-regulated in several types of tumors including colon cancer and this protein is normally expressed in the epithelial cells of the human colon. Therefore, compounds which enhance CD26 levels are expected to have antitumor potential.

It has been shown that apigenin alone is able to cause an increase of 56.3% in the cell surface abundance and activity of CD26 in different CRC cell lines (HT-29 and HRT-18), so some authors have studied whether this flavone is able to enhance the up-regulation in CD26 cell surface expression of irinotecan, 5-FU and oxaliplatin, that are three chemotherapeutic agents used for the treatment of colorectal cancer. In the case of 5-FU and oxaliplatin, no specific interaction was reported with the action of apigenin; however, the ability of apigenin to potentiate CD26 was much more robust when was combined with increasing concentrations of irinotecan, generating a 4.2-fold increase in the potency of this drug (with a reduction of EC_50_ for irinotecan from 4.68 to 1.26 μg/mL). An interaction was also observed when the experimental design was reversed, adding a fixed dose of irinotecan to a series of apigenin concentrations, increasing by 30 times the capacity of apigenin to enhance CD26 expression, lowering its EC_50_ from 32.8 to 1.10 μM. Therefore, it was observed that in the case of irinotecan (a topoisomerase I inhibitor), but not of 5-FU or oxaliplatin, there is a specific interaction with the action of apigenin due to a cross-talk in the mechanism of action of apigenin with irinotecan, as apigenin is able to inhibit toposimorease I-DNA complex which overlaps with the primary mechanism of action of irinotecan (Lefort and Blay, 2011). This presupposes that part of the mechanism of action of apigenin is intimately related to topoisomerases.

TRAIL (tumor necrosis factor-related apoptosis-inducing protein), a member of the TNF superfamily, is able to induce apoptosis through interaction with the death receptor 5 (DR5), whose expression is regulated by the tumor suppressor p53. TRAIL is not toxic in normal cells because non-neoplastic cells express high levels of decoy receptors (DcR) for TRAIL, which could interfere with TRAIL signaling; it shows acquired resistance in cancer cells (Almasan and Ashkenazi, 2003; Du et al., 2016).

Apigenin induces the expression of DR5 in a dose-dependent manner preventing the degradation of this protein by acting as a proteasome inhibitor and increasing its expression in the membrane, so this up-regulation of DR5 acts in a synergic form sensitizing to the treatment with exogenous soluble recombinant human tumor necrosis factor-related apoptosis-inducing ligand (TRAIL) in CRC DLD-1 cells, showing a greater apoptotic effect than the treatment with TRAIL alone. As said before, TRAIL is an attractive candidate for cancer therapy because it selectively induces apoptosis in cancer cells, and it has been shown that combination of TRAIL with apigenin did not induce expression of DR5 protein and enhanced TRAIL-induced apoptosis in normal human PBMCs cells (Horinaka et al., 2006). Therefore, the combined treatment of apigenin and TRAIL is a promising anticancer therapy.

Diverse anti-apoptotic proteins as Bcl-XL, Bcl-2, Bcl-w, and Mcl-1 can prevent cell death in tumor cells. ABT-263 (Navitoclax) is a novel oral inhibitor for Bcl-2 family proteins, acting as a Bcl-2 homology 3 (BH3) mimetic, and leading to apoptosis, except in tumor cells with Mcl-1 overexpression (Tolcher et al., 2015). As apigenin induces apoptosis in tumor cells by modulating different kinds of signaling pathways, including downregulation of Mcl-1 mRNA (Shi et al., 2015), this apigenin Mcl-1 downregulation may enhance the ABT-263 antitumor activity (Shao et al., 2013; Erdogan et al., 2016). Furthermore, the inhibitory effect of apigenin on ERK phosphorylation levels is significant when CRC HCT116 cells are cotreated with ABT-263 (Shao et al., 2013). All these findings were verified *in vivo* in a SCID mice model bearing HCT116 xenografts, in which treatment with ABT-263 or apigenin alone resulted in a 30% inhibition of tumor growth compared with untreated control, but this percentage was increased to 70% by combination therapy, with decreased Mcl-1 levels as well as phosphorylated prosurvival mediators ERK or AKT (Table 2; Shao et al., 2013).

Summing up, both apigenin and ABT-263 alone induced low apoptosis rates in HCT116, DLS1, SW48, HT29, and HCT-8 tumor cells, but in combination therapy, an increase of 80% in apoptosis was recorded via a caspase dependent mechanism. The combination index of these combinations was below 1.0, indicating a synergistic effect (Shao et al., 2013).

### Chrysin

Chrysin (Figure 1E) is another flavone found in honey, propolis, and various plant extracts such as chamomile and blue passion flower (*Passiflora caerulea*; Renuka et al., 2016). Chrysin has multiple biological activities, including antitumor effects in diverse cancer cell lines and tumor animal models (Kasala et al., 2015).

Several studies in SW480 CRC cells have shown that this flavone is able to induce cell cycle arrest at G2/M transition in a dose-dependent manner. Combination of chrysin plus apigenin doubled the proportion of SW480 cells in G2/M arrest; indicating that both flavones cooperate in slowing down tumor progression (Wang et al., 2004). *In vitro* studies on DLD1 CRC cells have demonstrated a chemoprotective effect of chrysin due to induction of AhR activity (Aryl Hydrocarbon Receptor) accompanied by p21 overexpression, a cell cycle important inhibitor (Ronnekleiv-Kelly et al., 2012). At early tumorigenesis stages, chrysin shows a chemopreventive activity by modulating normal cryptal cells proliferation and by activating apoptosis in aberrant cryptal cells (as those generated in an azoxymethane animal model for CRC). These activities are carried out by downregulating PCNA (Proliferating Cell Nuclear Antigen) and growth factors such as IGF-1 (Miyamoto et al., 2006, 2010; Kasala et al., 2015).

Tumor necrosis factor-alpha (TNF-α) is a pro-inflammatory cytokine with a wide range of biological activities also including both cell progression and death. These TNFα conflicting activities rely on TNF receptor 1 (TNFR1) activation of two different pathways: a caspase cascade for induction of apoptotic events, and nuclear transcription factor kappa-β (NF-kβ), which is a cell survival mechanism. Generally, most tumor cells are refractory to TNFα-induced apoptosis if they keep a working NF-kβ pathway (Chen and Goeddel, 2002; Karin et al., 2004). But chrysin sensitizes HCT116 CRC cells (highly resistant to TNFα apoptosis induction) toward TNFα-induced apoptosis due to its blocking of NF-kβ/caspase 8 pathway, by downregulating its trigger, c-FLIP-I (Li et al., 2010). Combination therapy with chrysin and TNFα together showed a 40% increase in cell death compared to monotherapy, due to caspase 8 activation (Table 2; Chen et al., 2004; Romier et al., 2008; Li et al., 2010).

TRAIL binding to death receptors as DR5 results in adaptor protein FADD (Fas-associated protein with death domain) and procaspase 8 or 10 recruitment, which then activate this death pathway. The main negative regulator of this pathway is the cellular caspase-8 (FLICE) inhibitory protein (c-FLIP). Its overexpression causes resistance to this apoptotic process, thereby limiting the therapeutic use of TRAIL. In order to overcome these resistances, a combination with TRAIL pathway sensitizers targeting c-FLIP expression may be a promising approach. In this sense, chrysin is able to suppress c-FLIP expression and to enhance DR5 expression in HT-29 cells, enhancing TRAIL-induced cell death in CRC cells (Ding et al., 2012).

Flavonoids antioxidant activity can also reduce chemotherapy side effects. Cisplatin generates a wide variety of ROS that interact with DNA, lipids, and proteins in CRC cells, including Pt-DNA adducts that hinder cell division and DNA synthesis/repair, leading to apoptotic events (Dasari and Tchounwou, 2014). In this sense, the prophylactic effect of chrysin against colon toxicity due to cisplatin was tested in Wistar rats, confirming a protective effect by reducing oxidative stress (Khan et al., 2012). Although, cisplatin is not used in CRC patients' treatment, these experiments in rats show interesting effects of chrysin with respect to ROS inducing agents.

With a similar action to cisplatin, vanadium compounds are considered a new class of non-platinum metal compounds with eventual low toxicity, although they are not used in clinical praxis. *In vitro*, these compounds inhibit cell cycle even at low doses, by generating ROS, which leads to DNA cleavage and apoptosis. Chrysin complexation with vanadyl cation increased antitumor activity in HT-29 cells (cell cycle arrest in G2/M transition) compared to the monotherapy treatment. Chrysin vanadate complex reduced to 56% the HT-29 cell survival vs. 88% in the case of cisplatin. This chrysin potentiating effect may be due to a reduction in GSH levels, one of the most important antioxidant defenses in mammals (León et al., 2015). In order to avoid gastrointestinal damage in preclinical trials, different vanadium complexes have been generated with flavonoids (Evangelou, 2002).

### Scutellarin

Scutellarin is a glycoside of the flavone scutellarein (Figure 1F), isolated from the traditional Chinese medicine plant *Scutellaria barbata* (Xing et al., 2011). It has been used in HCT116 cells as chemosensitizing agent (at 100 μM) combined with resveratrol (at 200 μM) and 5-FU (at 500 μM). These experiments showed an increase in apoptosis, due co caspase 6 activation, which was absent in p53 (−/−) versions of this cell line (Chan et al., 2009).

### Oroxylin

Oroxylin A is a O-methylated flavone (Figure 1G) extracted from the herb *Scutellariae radix*. Oroxylin A inhibits iNOS and COX-2 gene expression by blocking NF-κB. Also, this flavone inhibits LPS-induced NF-kB activation by blocking IκB degradation, the protein which usually binds NF-kB in the cytosol, keeping it in its inactive form (Chen et al., 2000).

Combination of oroxylin A with 5-FU (1:5) both *in vivo* and *ex vivo* in a CRC model using HT-29 cells showed a synergistic action, with COX-2 inhibition and increased ROS generation, which led to HT-20 sensitization to 5-FU. 5-FU IC_50_ in HT-29 is 4.63 mmol/L, but when combined with oroxylin A, this value diminishes to 764 μmol/L. To corroborate this synergistic effect, in a nude mice xenograft model for HT-29, 100 mg/kg oroxylin A plus 20 mg/kg 5-FU showed a 66% tumor size decrease, in comparison with 36 and 42% reduction in the monotherapies, respectively (Table 2; Ha et al., 2012). Therefore, oroxylin combination therapy could be a valuable tool in order to reduce 5-FU doses and subsequent *in vivo* side effects.

### Kaempferol

Kaempferol (Figure 1H) is a flavonol present in black tea, broccoli, propolis, grapefruit, and other plant sources. This compound has a marked antitumor potential on different types of cancer cells (Gutiérrez-del-Río et al., 2016). In CRC cells, it induces p53-dependent growth inhibition and, at the same time, apoptosis by inducing cytochrome c mitochondrial release and caspase-3 cleavage activation (Li W. et al., 2009; Lee H. S. et al., 2014b). In HT-29 cells, this flavonol induces apoptosis and inhibits IGF-IR and ErbB3 signaling (Lee H. S. et al., 2014a). Kaempferol is also able to induce G1 and G2/M cell cycle arrest by inhibiting the activity of CDK2, CDK4, and Cdc2 (Cho et al., 2013).

Monotherapy with kaempferol or TRAIL alone showed a slight effect on apoptosis induction in SW480 and DLD-1 CRC cells, while the combination therapy induced a dramatic apoptosis increase in a kaempferol dose-dependent manner. This means that kaempferol is able to sensitize these CRC cells to TRAIL-induced apoptosis. Interestingly, this combination of drugs showed very low cytotoxicity in PBMC normal cells (Yoshida et al., 2008).

### Quercetin

Quercetin (Figure 1I) is an ubiquitous flavonol in nature, where it is found in onion, apples, and many other vegetables and fruits. Quercetin inhibits RASA1 expression in CRC cell lines, avoiding RAS activation and therefore its proliferative effects (Ranelletti et al., 2000). Quercetin was combined with TRAIL for treatment of three CRC cell lines, Caco-2 (adenoma), SW-620, and HT-29 (adenocarcinomas); demonstrating that this flavonol is a potent sensitizer to TRAIL-induced apoptosis in a synergistic manner (SW-620 and HT-29), whereas this combination resulted in an additive effect in the case of adenoma cells (Caco-2). These pro-apoptotic effects seem to be associated with a membrane distribution of quercetin in lipid rafts domains, regions which are rich in cholesterol, sphingolipids, and TRAIL death receptors. This membrane distribution could be the initiator for signal cascades causing TRAIL-mediated apoptosis (Psahoulia et al., 2007).

Quercetin has been also used in combination with 5-FU *in vitro*, treating CO115 (p53 positive) and HCT15 (p53 negative) CRC cell lines. This combination of drugs showed higher apoptosis levels in CO115 cell line, in a synergistic manner, but an additive effect in HCT15 cells. This enhanced apoptosis was even higher than with 100 times higher 5-FU concentration in monotherapy. p53 may elicit this synergistic pro-apoptotic effects by enhancing caspase 3 activation and diminishing Bcl-2 (anti-apoptotic) levels. This involvement of p53 is reinforced when a siRNA is used to silence p53 expression in CO115 cells, losing the synergistic effect of this combination of drugs (Table 2; Xavier et al., 2011).

### Epigallocatechin

(-)-Epigallocatechin-3-gallate (EGCG, Figure 1J) is the major polyphenolic constituent of green tea, representing 200–300 mg/brewed cup (Singh et al., 2011). The antitumor effects of this and other flavanols are widely supported by epidemiological, *in vitro*, animal and clinical studies (Singh et al., 2011). For example, different concentrations of grape seed extracts [rich in (-)-epicatechin] were tested in combination with 5-FU 100 μM in Caco-2 cells, showing a slightly synergistic effect on cell apoptosis (Cheah et al., 2014).

EGCG and related compounds are able to inhibit several critical signal transduction pathways in cancer cells. For example, EGCG inhibits multiple RTKs (Receptor Tyrosine Kinase) as the IGF/IGF1R system, EGFR, and HER2 receptors, which play key roles in CRC cell proliferation (Shimizu et al., 2005; Adachi et al., 2008, 2009). EGCG also blocks cell proliferation and cell migration in CRC cells, by inhibiting the signaling pathway TF (Tissue Factor)/VIIa/PAR2 (Protease-Activated Receptor 2) that usually mediates ERK1/2 phosphorylation and final activation of the pro-inflammatory NF-kβ. This lower activity of the transcription factor NF-kB induces an up-regulation of caspase-7 and a down-regulation of MMP-9 matrix metalloprotease expression, affecting proliferation and migration of tumor cells (Table 2; Zhou et al., 2012).

Furthermore, EGCG is an epigenetic regulator, which contributes to degradation of DNMT3A (DNA Methyltransferase 3A) and HDACs (Histone Deacetylases) through a process of ubiquitination in CRC cells sensitive to methylation. These effects, together with histones deacetylation, are very important epigenetic mechanisms in tumorigenesis, as they are responsible for silencing various tumor suppressor genes and other ones involved in cell cycle regulation and apoptosis (Moseley et al., 2013). Therefore, EGCG is able to restore the expression of genes involved in tumor suppression such as RXRalpha (Retinoid X Receptor alpha) that are silenced by epigenetic processes in tumor cells (Morris et al., 2016).

Also, it is remarkable the effect exerted by EGCG at the level of CSCs, downregulating *Notch* signaling, a membrane receptor, which is directly involved in the differentiation and proliferation of CRC stem cells (Jin et al., 2013).

Based on these anti-proliferative, anti-metastatic and epigenetic activities for EGCG, its effectiveness in combination with anticancer drugs has been tested. The combination of 5-FU with EGCG resulted in a synergistic growth inhibition in several human CRC cell lines (DLD-1, SW480, and COLO201) (Kumazaki et al., 2013). The same combination on HT-29 and HTC-116 CRC cells reduced cell viability significantly compared with the monotherapies. The EGCG potentiating effect on the 5-FU is supposed to be due to a downregulation in the expression of ABC transporters, which causes higher intracellular 5-FU concentrations (Hwang et al., 2007b; Wubetu et al., 2015).

Sodium butyrate is a non-toxic compound naturally produced in the colon after microbial fermentation of dietary fiber, which shows strong antitumor effects only on transformed colonocytes. Combination of EGCG and sodium butyrate on HT-29 tumor cells caused a synergistic reduction in survivin protein and mRNA levels, an anti-apoptotic protein highly expressed in CRC (Saldanha et al., 2014).

Another compound inducing apoptosis in tumor colonocytes, sulindac, is a NSAID inhibiting COX-1 (expressed constitutively in all tissues) and COX-2 (highly expressed in CRC and induced by cytokines). Although, sulindac has side effects due to this broad cyclooxygenases inhibition, its combination with EGCG reduces them due to an enhanced inhibition of COX-2 (Suganuma et al., 1999). This synergistic effect has been also observed in rats developing CRC by induction with the chemical inducer azoxymethane (Ohishi et al., 2002).

### Genistein

Genistein (Figure 1K) is an isoflavone which can be found in high concentrations in soybeans, lentils, beans, and chickpeas. Numerous epidemiological studies have reported a negative correlation between the incidence of CRC and diets rich in soybean (Spector et al., 2003; Rossi et al., 2006). This isoflavone has a growing interest as a pro-apoptotic agent because of its specific and almost exclusively activity against tumor cells (as CRC) rather than normal ones (Marín et al., 2015). Genistein acts by increasing the expression of pro-apoptotic proteins as Bax or p21 (Yu et al., 2004), by inhibiting NF-kβ (Luo et al., 2014) and topoisomerase II (Mizushina et al., 2013), by regulating ERB expression (Pampaloni et al., 2014), by suppressing the carcinogen induction of WNT/β-catenin signaling pathway (Zhang et al., 2013), by increasing the expression of antioxidant enzymes such as glutathione peroxidase (Ganai and Farooqi, 2015), and by preventing human CRC metastases due to MMP2 metalloproteases inhibition (Xiao et al., 2015). All these activities can be exploited by combinatory approaches in order to prevent or to treat CRC.

As it has been mentioned above, 5-FU is widely used in the treatment of solid tumors such as CRC, but its main clinical limitation is the development of resistant phenotypes by the over-expression of anti-apoptotic proteins or cell proliferation factors. In order to overcome this resistance problems, a combinated treatment with genistein on HT-29 cells resistant to 5-FU showed a significant reduction in cell viability compared to the monotherapy. These experiments demonstrated a synergistic effect on cell growth inhibition by over-expression of pro-apoptotic *p53* and *p21* genes and downregulation of survival genes such as *Glut-1*. However, the main mechanism involved in this combination was due to COX-2 expression inhibition (Hwang et al., 2005). In a similar way, combination of genistein with cisplatin also inhibited cell growth and induced apoptosis in a synergistic manner in HT-29 CRC cells, by inhibiting tyrosine kinases (Hu et al., 2014). Finally, combination with dexamethasone also shows synergistic effects by increasing p21 levels in Colo320 HSR cells, inhibiting their growth (Park et al., 2001).

Together with chemotherapy, radiotherapy also plays a crucial role in the treatment of rectal cancer, however, in more than 70% of patients, it causes side effects on the gastrointestinal system, as mucositis due to the generation of free radicals by ionizing radiation, which causes oxidative damage to normal colonocytes. A solution to this problem would be to combine radiotherapy with natural radioprotective agents (Jagetia, 2007). In this sense, the remarkable antioxidant activity of genistein, combined with its ability to activate antioxidant pathways, makes it a perfect candidate to protect against radiation cellular damage. Following this hypothesis, CT26 CRC cells were injected into BALB/c mice, and animals were treated with radiotherapy in the abdominal area. After a combination with genistein, this isoflavone reduced the apoptosis in normal cells and improved morphological changes in healthy intestinal mucosa. Also, tumors size was lower in mice subjected to combination therapy (Table 2; Son et al., 2013).

The epidermal growth factor receptor (EGFR) plays a very important role in tumor progression because binding of its ligands initiates a cascade of intracellular phosphorylations that ultimately triggers genes associated with cell proliferation, survival, or invasion. This receptor is over-expressed in tumor cells and diverse drugs inhibit this tyrosine kinase, although resistance phenotypes usually appear after irradiation (Singh et al., 2016). Interestingly, pretreatment with genistein during 24 h before irradiation was able to perform a synergistic effect on irradiated HTC116 cells survival (CI < 0.7), due to an enhanced EGFR inhibition (Gruca et al., 2014).

### Silymarin

Silymarin is a flavolignan extract from milk thistle (*Sylibum marianum*). This flavolignans mixture contains silibinin (silybin A and B, the most active compounds, Figure 1L), isosilybin (A, B), silydianin, and silychrystin (Lee et al., 2006). Silybins induce cell cycle arrest and apoptosis by acting on cyclin dependent kinases (CDKs).

Silymarin has been tested in combination with doxorubicin and paclitaxel against CRC cells, in a cell line sensitive to doxorubicin (LoVo) and its multidrug resistant isogenic version (LoVo/DX). Twenty-four hour prior to treatment with both drugs, silymarin was used in these cells, showing a synergistic effect in LoVo cell line, but not in LoVo/DX cells, where an additive effect was observed. This additive effect may be valuable when dealing with *in vivo* experiments, as any contribution to reduce doxorubicin doses would also reduce its side effects, mainly associated to cardiotoxicity (**Figure 3**). This cardiotoxicity is due to formation of iron-related free radicals, as well as damages to mitochondrial NAD(P)H oxidase complex (Thorn et al., 2011). Silymarin causes higher intracellular drug concentrations in LoVo cells due to a repression of P-gp pump (P-glycoprotein, MDR1). However, in LoVo/DX cells, the strong P-gp overexpression prevents this sensitizing effect (Colombo et al., 2011).

Silibinin also enhances metformin antiproliferative effects. Combination of this antidiabetic agent at 10 mmol/L plus 100 μmol/L of silibinin in COLO205 CRC cell line showed a synergistic inhibition of 60% in cell survival, which did not affect normal HCoEpiC cells. Monotherapy at these concentrations had no effects. This combination of drugs increased caspase 3 activation and AIF expression, resulting in apoptosis activation by extrinsic and mitochondrial ways. A role for the PTEN/Akt pathway in this apoptosis induction in cancer cells was also observed, with increased PTEN levels and decreased phosphorylated protein kinase B (p-Akt) (Table 2; Tsai et al., 2015).

Vanadium (IV) complexes have been tested in combination with silibinin and chrysin against HT-29 cell line, showing increased cytotoxic effects at 100 μM vanadyl ion in comparison with monotherapies (280 μM vanadyl). In this combination, chrysin induces cell cycle arrest in G_2_/M transition, while silibinin induces apoptosis due to caspases activation and NF-κB inhibition (León et al., 2015).

### Flavopiridol

Flavopiridol (Alvocidib, Figure 1M) is a semi-synthetic flavonoid-like derivative, generated from rohitukine, an alkaloid from the bark of *Dysoxylum binectariferum*, a tree from India (Kelland, 2000). Flavopiridol inhibits cyclin-dependent kinases (CDKs), targeting the ATP-binding pocket of their catalytic subunit, as Cdk1, Cdk2, Cdk4, Cdk6, Cdk7, and Cdk9. Also, it inhibits other kinases like PKA, PKC, Erk-1, EGFR, and other receptor associated protein kinases. Flavopiridol blocks cell cycle in G_1_/S and G_2_/M transitions by lowering expression levels of cyclin D1, p27^Kip1^, and p21^Waf1/Cip1^. Low cyclin D1 levels cause a reduction in Cdk4 concentration, leading to an accumulation of hypophosphorylated retinoblastoma protein (Rb), which causes cell cycle arrest. Low p27^Kip1^ or p21^Waf1/Cip1^ levels also cause a reduction in Cdk2 concentration, inducing cell cycle arrest in G_1_phase and inhibiting EEF1B2 elongation factor, which blocks RNApol II transcription of Newcomb (2004).

Flavopiridol is a potent apoptosis inducer in tumor cells, via the mitochondrial pathway (release of cytochrome c or caspases activation), but also via AIF (apoptosis-inducing factor) pathway (Achenbach et al., 2000). This pro-apoptotic effect is enhanced as flavopiridol also inhibits Akt activation, leading to NF-κB inactivation and therefore to an inhibition of proliferation processes (Takada and Aggarwal, 2004). Therefore, this flavonoid derivative triggers apoptosis and at the same time inhibits proliferation.

Irinotecan, a semisynthetic analog of the natural alkaloid camptothecin, and SN-38, the irinotecan bioactive metabolite, prevents DNA from unwinding by inhibiting topoisomerase 1. Combination of SN-38 treatment followed by flavopiridol in HCT116 cell line and its null isogenic p53 (−/−) equivalent showed apoptosis induction only in the p53 wild type cell line. Here, p53 produced Rad51 mRNA downregulation, a gene coding for a DNA-repair protein (Ambrosini et al., 2008).

In a mouse xenograft model with HCT116 cell line, irinotecan treatment followed by flavopiridol showed a significant decrease in tumor growth compared to monotherapies. This study suggests changes in the choline kinase activity and decreased phosphocholine (Motwani et al., 2001; Darpolor et al., 2011). Since diarrhea is one of the most common side effect associated to irinotecan treatment, this synergistic effect among flavopiridol and irinotecan may be a valuable combination for preventing or reducing this gastrointestinal toxicity associated to this camptothecin derivative (Fuchs et al., 2003) (**Figure 3**).

Sequential treatment with docetaxel, flavopiridol, and 5-FU in HCT116 cell line showed an 8-fold increase in caspase activity, with much lower increase if the three compounds were added simultaneously, in pairs or separately. Using this triple combination in mice xenografts with HCT116 caused a decrease in tumor volume by 95% (50% reduction for single drug treatment, 70% reduction for two drugs combination; Table 2; Guo et al., 2006).

A phase I trial of weekly, sequential docetaxel followed by flavopiridol (after 4 h first treatment) in patients with advanced solid tumors showed that this combination of drugs was well tolerated, with one dose-limiting toxicity occurring at 70 mg/m^2^ flavopiridol. Docetaxel common toxicity effects are mostly associated to neutropenia (Ho and Mackey, 2014). Also, one complete response was observed in a patient with pancreatic carcinoma, as well as four partial responses in pancreatic (1), breast (2), and ovarian (1) tumors. Stable disease was observed in ten patients (27 patients in total; Fornier et al., 2007).

## Terpenes

Another large and diverse class of organic compounds where some of them have shown a promising role against CRC in combination with other drugs are the terpenes. Terpenes are the structurally most diverse class of all plant and fungal bioactive metabolites, with more than 50,000 molecules. All terpenes derive from the condensation of dimethylallyl diphosphate (DMAPP) and isopentenyl diphosphate (IPP) precursors, which may be linked together “head to tail” to form linear chains or may be arranged to form rings (Klein-Marcuschamer et al., 2007). Terpenes are classified, according to the number of biosynthetic isoprene units, in monoterpenes (10 carbon atoms, C10), sesquiterpenes (C15), diterpenes (C20), triterpenes (C30), and tetraterpenes (C40) (Misawa, 2011). In addition, terpenes that have undergone oxidation steps are called terpenoids. Some examples are essential oils such as limonene (C10, flavoring agent), vitA (C20), β-carotene (C40), and steroids (C30, cholesterol, testosterone).

### Artesunate

**Figure 2:**
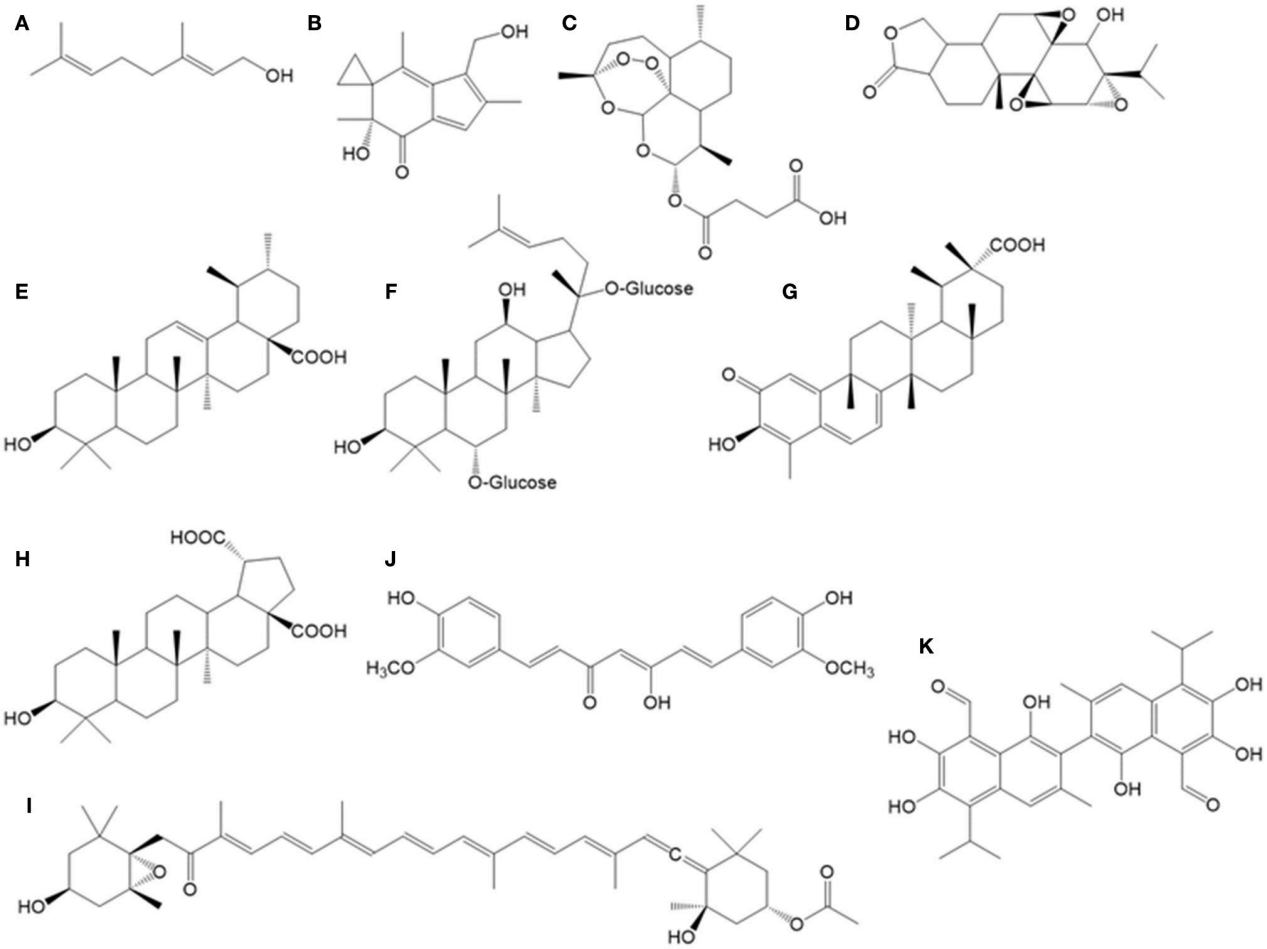
**Chemical structures of bioactive terpenoids and other compounds described in the text. (A)** geraniol, **(B)** irofulven, **(C)** artesunate, **(D)** triptolide, **(E)** ursolic acid, **(F)** ginsenoside, **(G)** celastrol, **(H)** betulinic acid, **(I)** fucoxanthin, **(J)** curcumin, **(K)** gossypol.

Artesunate (Figure 2C) is the hemisuccinate ester of artemisin, a sesquiterpene found in *Artemisa annua* (a traditional Chinese herb), widely used for malaria treatment as ROS inducer in the *Plasmodium* parasite (Meshnick, 2002). Artesunate is cytotoxic in HCT116 cells, inducing a cell cycle arrest at G_1_, due to cyclin D1 downregulation and p21 overexpression. The treatment of these CRC cells with artesunate (1.9 μM) or oxaliplatin (another agent also causing ROS stress in cells, together with other alkylating activities on DNA; 4 μM) causes 50% cell killing. However, the same effect can be obtained with a combination of just 0.65 μM artesunate plus 1.6 μM oxaliplatin, which reinforces the used of artesunate as a possible adjuvant chemotherapy molecule (Liu et al., 2011).

### Geraniol

The monoterpene geraniol (Figure 2A) is a main component in commercially important essential oils (rose, lemon, etc.), with wide use in perfumes. *In vitro* combination of geraniol (150 μM, IC_30_) plus 5-FU (0.25 μM, IC_30_) on Caco-2 cells increased the cell death in comparison with monotherapy, causing over 20% reductions cell survival. In a tumor xenograft model for TC118 CRC cell line, combination of geraniol (150 mg/kg) plus 5-FU (40 mg/kg) showed a clear 83% reduction in tumors size, whereas monotherapies with these concentrations caused only 26 and 30% tumor reductions, respectively (Carnesecchi et al., 2004).

In a CRC animal model using the mutagen dimethylhydrazine, oral geraniol (25 mg/100 g) was administered in order to test its effects in CRC prevention at early stages (aberrant crypt foci reduction in colon mucosa, ACF). After 9 weeks, a significant 37% reduction in colon ACF was observed with respect to control animals, together with enhanced apoptosis parameters. This protection was also accompanied by a 30% reduction in the cellular levels of Bcl-2 in geraniol treated animals, an anti-apoptotic protein, whose low levels may explain the geraniol antitumor effect in colon mucosa (Table 3). These results may open the path to the study of other non-cyclic monoterpenes as antitumor agents (Vieira et al., 2011).

**Table 3:** **Summary of main ***in vitro*** and ***in vivo*** synergistic effects of terpenoids and other compounds in combination with chemotherapeutic compounds against CRC**.

**References**	**Tested molecule**	**In combination with**	**Experimental model**	**Main result**	**Proposed mechanism**
Carnesecchi et al., 2004; Vieira et al., 2011	Geraniol	5-FU	Caco-2 cell line	Synergistic: 20% reduction in cell survival	Down-regulation of Bcl-2
Carnesecchi et al., 2004	Geraniol	5-FU	Mice xenograft (TC118)	Synergistic: 80% reduction in tumor size	Unknown
Serova et al., 2006	Irofulven	Oxaliplatin	HT-29 cell line	Synergistic: reduced cell survival	Unknown
Britten et al., 1999	Irofulven	Irinotecan	Mice xenograft (HT-29)	Synergistic: tumor size reduction	Unknown
Liu et al., 2011	Artesunate	Oxaliplatin	HCT116 cell line	Synergistic: 50% cell killing	ROS induction
Liu et al., 2014	Triptolide	Oxaliplatin	SW480 cell line	Synergistic: 62% cell killing	Apoptosis induction, blocking of β-catenin translocation to nucleus
Liu et al., 2014	Triptolide	Oxaliplatin	Mice xenograft (SW480)	Synergistic: 60% tumor growth reduction	Unknown
Koh et al., 2012; Prasad et al., 2012	Ursolic acid	Radiotherapy	CT26 and HCT116 cell lines	Synergistic: 55% cell killing	Apoptosis induction, caspase 3 activation, ROS increase, GSH, NF-kB and Bcl-2 reductions
Wang et al., 2015	Ginsenolides	5-FU	Mice xenograft (HCT116)	Synergistic: reduced tumor size	G_1_ arrest
Kim et al., 2009	Ginsenolides	Docetaxel	HCT116 cell line	Synergistic: increased cell death	NF-kB inhibiton, Bcl-2 repression
Zhu et al., 2010	Calastrol	TRAIL	SW620 cell line	Synergistic: increased cell killing	Apoptosis induction
Jung et al., 2007	Betulinic acid	5-FU, oxaliplatin, irinotecan	SNU-C5 cell line	Synergistic: increased cell killing, reduction in chemoresistance	Apoptosis induction (caspase 3)
Li et al., 2007	Curcumin	Oxaliplatin	Lo-Vo cell line	Synergistic: growth inhibition	Unknown
Anitha et al., 2014	Curcumin	5-FU	HT-29	Synergistic: increased cells killing	Apoptosis induction
Murakami et al., 2013	Curcumin	Turmerones	CRC mouse model (dimethyl-hydrazine)	Synergistic: tumor size reduction	Apoptosis induction
Yue et al., 2016b	Curcumin	Bevacizumab	Mice xenograft (HT-29)	Synergistic: tumor size reduction	Apoptosis induction
Zhang et al., 2003; Lan et al., 2015	Gossypol	5-FU	Mice xenograft (HT-29)	Synergistic: tumor size reduction	Apoptosis induction, chemical sensitization

### Irofulven

Irofulven (Figure 2B) is a semi-synthetic derivative of illudin S, a sesquiterpene isolated from the mushroom *Omphalotus illudens*. It shows potent growth inhibition on a wide variety of human solid tumor cell lines and primary tumor cell types. *In vivo* testing has demonstrated excellent dose-related antitumor activity in several human tumor mice xenograft models.

Combination of irofulven with radiation or chemotherapeutic agents such as paclitaxel, irinotecan, 5-FU, mitomycin C, thiotepa, topotecan, and cisplatin have produced additive and/or synergistic inhibition of cellular proliferation in a variety of tumor types. With respect to CRC, simultaneous exposure to irofulven and cisplatin is at least additive for HCT116 cells, whereas simultaneous exposure to irofulven and 5-FU is additive for HT-29 cells and synergistic for the irofulven-resistant HCT116 cell line (Poindessous et al., 2003). Combination of irofulven with oxaliplatin also led to synergistic activity in HT-29 cell line (Serova et al., 2006). In a mice xenograft model for HT-29 cells, combination of irofulven and irinotecan, significant reduction in tumor weights occurred with partial responses in nearly all of the animals and some animals achieving complete responses (Table 3; Britten et al., 1999).

### Triptolide

Triptolide (Figure 2D) is a diterpene from *Tripterygium wilfordii* tree, used as anti-inflammatory and antitumor in traditional Chinese medicine. Individual treatments with triptolide or oxaliplatin during 48 h in SW480 cell line showed IC_50_ values of 16.7 ng/mL and 20.8 μg/mL, respectively. However, in combination with 10 μg/mL oxaliplatin, 8 ng/mL of this diterpene was able to induce 62% apoptosis. This synergistic effect was due to an inhibition of nuclear translocation of the transcription factor β-catenin under combinatory conditions, causing that this cell progression factor remains accumulated in the cytoplasm. Also, in a mice xenograft model for this cell line, combination of triptolide (0.1 mg/kg) with oxaliplatin (5 mg/kg) also showed this synergistic effect, reducing tumors growth by 60% (Table 3). This positive effect was not accompanied by a significant increase in ALT and AST transaminases (biomarkers for hepatic damage) nor in blood urea nitrogen (biomarker for renal damage; Liu et al., 2014). In a similar way, combination of triptolide (0.15 mg/kg) plus 5-FU (12 mg/kg) in another xenograft model with HT-29 cell line caused a reduction of 96% tumor growth for 3 weeks treatment, with no side effects observed (Tang et al., 2007). These results open the way for the use of triptolide in the treatment of solid tumors in preclinical trials (Jiang et al., 2001; Fidler et al., 2003).

### Ursolic acid

The triterpene ursolic acid (Figure 2E) is found in diverse herb species as basil and rosemary. This antioxidant compound is able to modulate cellular redox status in normal cells, but in tumor cells it exerts pro-oxidative action. This is an important fact when dealing with tumor cells and radiotherapy, as ionizing radiation works by increasing cell oxidative damage in transformed cells, leading to apoptosis. In this sense, radio-resistance status can be avoided or reverted by using drugs able to increase ROS and its mitochondrial, DNA and membranes damages.

Using *in vitro* experiments with CT26 mouse CRC cells, a synergistic effect has been described for a combination of ursolic acid plus radiotherapy, where apoptosis was enhanced 55%, via caspase 3 activation. In these co-treated tumor cells, it was observed higher peroxides formation, lower GSH levels and extended mitochondrial damage (Koh et al., 2012). These pro-apoptotic effects of ursolic acid have been reproduced in other CRC cell lines as HCT116, where this compound was able to reduce the pro-inflammatory NF-kB cytokine, the pro-metastatic MMP-9 matrix metalloprotease, and the survival effectors Bcl-2 and survivin. All these changes in expression of key cancer modulators were reinforced when conducting these *in vitro* experiments with ursolic acid and capecitabine together (Table 3; Prasad et al., 2012).

In a mouse xenograft model with HCT116 cells, combination of ursolic acid with capecitabine caused a 68% reduction in tumor volume, also diminishing distant metastasis to lung around 60% (Prasad et al., 2012).

### Ginsenosides

Panaxadiol is a ginsenosides (Figure 2F) triterpene found both in ginseng (*Panax ginseng*) and in notoginseng (*Panax pseudoginseng*). Previous studies have shown the antitumor activities of these compound on several cell lines and their targeting on multiple cancer signaling pathways (Park et al., 1999; Jin et al., 2003; Gao et al., 2013). Notoginseng extract, which contains high amounts of ginsenosides, enhances 5-FU induced apoptosis in human CRC cells (Wang et al., 2007a,b). Looking for specific bioactive components in these extracts, panaxadiol was found to be the component which caused enhanced apoptosis in HCT116 cell line (Li X. L. et al., 2009). Similar results were obtained with protopanaxadiol, another ginseng metabolite that significantly enhanced 5-FU effects on HCT116 cells by inducing arrest in G_1_ phase and apoptosis. These *in vitro* data were confirmed by using an *in vivo* mice xenograft model, showing that protopanaxadiol and 5-FU co-administration very significantly reduced the tumor size in a dose-related manner (Table 3; Wang et al., 2015).

Another member of this family, ginsenoside Rg3, is able to repress NF-kB expression in HCT116 cell line, leading to apoptosis, with a IC_50_ value of 100 μM. NF-kB is usually activated in these and other tumor cells. Twenty-four hour combination of 50 μM ginsenoside R3 plus 5 μM docetaxel in this cell line resulted in a synergistic NF-kB inhibition, absent in the monotherapy experiments at these concentrations. This pro-apoptosis effect was due to Bcl-2 repression and expression of the pro-apoptotic proteins caspase 3 and Bax, which demonstrated the chemosensitization of these tumor cells to docetaxel in the presence of ginsenosides R3 (Kim et al., 2009).

### Celastrol

Celastrol (Figure 2G) is a triterpene from the bark of the *T. wilfordii* tree. This compound inhibits the heat shock protein HSP90, blocking its interaction with Cdc37. TRAIL addition to cultures of SW620 CRC cells shows an IC_50_ value of 423.5 ng/mL, whereas this parameter is reduced to only 121.1 ng/mL in the presence of 2 μM celastrol during 72 h, due to apoptosis induction via caspase 3; therefore, celastrol shows a synergistic effect in combination with TRAIL (Zhu et al., 2010).

### Betulinic acid

Betulinic acid (Figure 2H) is an anti-inflammatory and antimalarial triterpene isolated from diverse plants, as *Betula pubescens* tree (birch) and many others. This compound exerts apoptosis through caspase 3 induction in SNU-C5 CRC cell line with IC_50_ value of 1 μg/mL. Three resistance variants were originated from this cell line, showing increased resistance to 5-FU (total resistance, instead IC_50_ of 6 μg/mL in parental cell line), irinotecan (5.55-fold IC_50_ instead of IC_50_ of 18 μg/mL in parental cell line), and oxaliplatin (total resistance, instead IC_50_ of 100 μg/mL in parental cell line). These three resistant variants were more sensitive to betulinic acid alone than the parental cell line, but more interestingly, combination of betulinic acid plus 5-FU reverted apoptosis induction in the 5-FU resistant cells. A similar reversion effect was observed with a combination of betulinic acid plus oxaliplatin in oxaliplatin-resistant cells. These results clearly demonstrate that in some cases it is possible to circumvent acquired chemoresistance by combination therapy of anticancer drugs with chemosensitizers as betulinic acid (Jung et al., 2007).

### Fucoxanthin

Fucoxanthin (Figure 2I) is a tetraterpenoid carotenoid found in the edible macroalga *Undaria pinnatifida*, which has been associated to prevention of CRC (Kim et al., 1998). *In vitro* studies with Caco-2 cell line have shown that this carotenoid is able to induce apoptosis after 72 h exposure at 22.6 μM. This apoptosis induction was due to an 80% reduction in Bcl-2 protein levels, a survival factor (Hosokawa et al., 2004).

## Curcumin

Curcumin (Figure 2J) is a diarylheptanoid found in turmeric (*Curcuma longa*) that is frequently described as a chemopreventive agent for CRC (Chauhan, 2002; Goel et al., 2008; Prasad et al., 2014). Curcumin protects against chemically induced intestinal tumorigenesis in mice and rats (Huang et al., 1992, 1994; Rao et al., 1993; Kim et al., 1998; Kawamori et al., 1999) and prevents adenoma development in the gastrointestinal tract of *apc*(±) mice, a model of human familial adenomatous polyposis (Perkins et al., 2002).

A combination of liposomal curcumin with oxaliplatin *in vitro* at equimolar concentrations resulted in no significant enhanced growth inhibition compared with monotherapies results. However, 4:1 molar ration combinations in LoVo cells resulted in a synergistic effect. However, there was no synergistic effect for both drugs *in vivo* using Colo205 and LoVo mice xenografts (Li et al., 2007).

Dasatinib is a potent Src and Abl kinases inhibitor. Curcumin showed synergistic effect with this inhibitor in HCT116 and HT-29 cells under FOLFOX treatment (5-FU, leucovorin plus oxaliplatin) resistant phenotype. This combination of drugs is preferred to single agent regimens, as oxaliplatin alone, which has limited activity. FOLFOX inhibited cellular growth, invasion and colonosphere formation and also reduced CSCs populations as evidenced by the decreased expression of their specific markers (CD133, CD44, CD166, and ALDH; Nautiyal et al., 2011).

**Figure 3:**
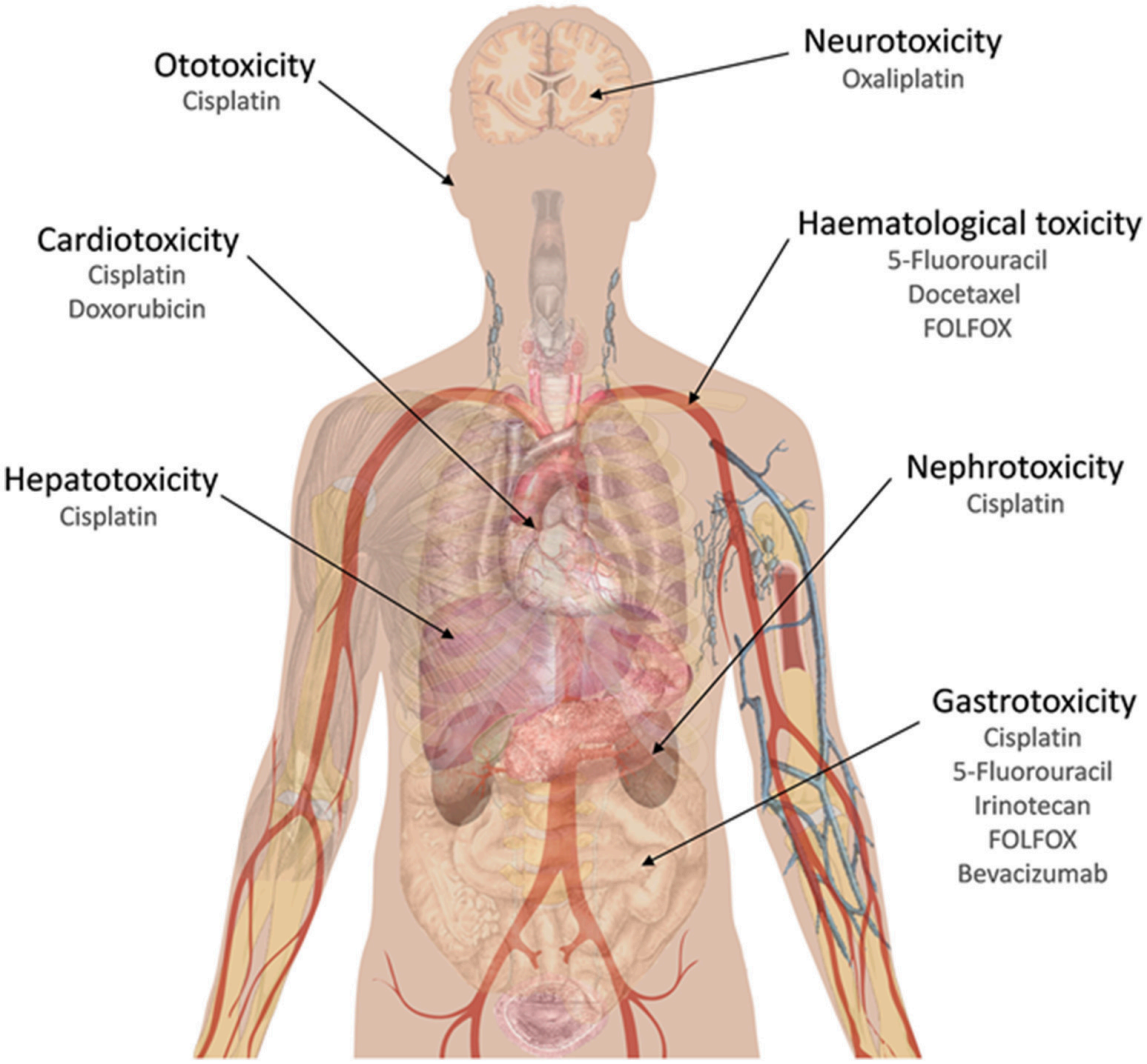
**Chemotherapy compounds that have been mentioned in this work and their main side effects**.

Different studies have also reported the potential enhancement of 5-FU antitumor efficacy in combination with curcumin/hexahydrocurcumin, both *in vitro* (Du et al., 2006; Srimuangwong et al., 2012a) and *in vivo* (Srimuangwong et al., 2012b). Curcumin can potentiate as well the pro-apoptotic and anti-metastatic effects of capecitabine, a prodrug that is enzymatically converted to 5-FU in the body (Kunnumakkara et al., 2009). In a recent phase I clinical trial using a combination of curcumin with FOLFOX, this combination of drugs enhanced anti-proliferative effects in patient-derived explants, indicating that curcumin can reduce CRC cells survival (Patel et al., 2008; James et al., 2015). Oxaliplatin treatment causes neurosensory toxicity and paresthesia, but combination therapy with FOLFOX regimen leads to common neutropenia, neurotoxicity, and diarrhea (Braun and Seymour, 2011) (Figure 3).

Curcumin is relatively safe for normal cells, but it can induce tumor apoptosis by different pathways (Hanif et al., 1997; Ravindran et al., 2009; Kantara et al., 2014), as it has demonstrated in several clinical trials (Bar-Sela et al., 2010; Gupta et al., 2013). However, its poor bioavailability is still regarded as a major problem for its therapeutic use (Anand et al., 2007). One approach to enhance curcumin absorption by colonocytes, to increase *in vitro* bioactivity and *in vivo* bioavailability is nanoencapsulation. This promising process reduces the non-selective exposure of this nutraceutical and improves the plasma half-life of the drug (Tsai et al., 2011; Yallapu et al., 2012). For example, 5-FU and curcumin were individually entrapped in chemically modified chitosan nanoparticles that were characterized for its *in vitro* hemocompatibility, drug release profile, cellular internalization, *in vitro* combinatorial antitumor effects in HT-29 cells and plasma concentration time profile by pharmacokinetics (in Swiss Albino mouse model). These experiments demonstrated that nanoparticles were blood-compatible, the release profile over a period of 4 days was sustained, the antitumor effects on CRC cells were enhanced and the plasma concentrations of both components in the mouse model were improved and prolonged up to 72 h, longer than bare drugs (Anitha et al., 2014).

A synergistic combination of curcumin and resveratrol has been also described. Both agents, acting together, inhibited the constitutive activation of EGFRs and IGF-1R in HCT-116 CRC cells. A test with a mice xenograft mouse model of CRC showed that the combination of resveratrol and curcumin (at doses of 50 and 500 mg/kg, respectively, administered by gavage for 3 weeks) is highly effective in inhibiting tumor growth and stimulating apoptosis of CRC cells *in vivo*, through attenuation of NF-κB activity (Table 1; Majumdar et al., 2009).

Turmerones are several structurally related non-polar sesquiterpenes found in turmeric ethanol extracts, which could increase curcumin accumulation inside colonocytes, but this curcumin-free fraction also exhibits biological activities. Pharmacokinetic results showed that plasma curcumin levels in mice fed with turmeric extract were the highest ones (Aggarwal et al., 2013; Yue et al., 2016b). Interestingly, the combination of curcumin and turmerones abolishes tumor formation when fed to a dimethyl-hydrazine-initiated and DSS-promoted mouse model of CRC (Murakami et al., 2013). Also, in a HT-29 tumor xenograft mice model, feeding with turmeric ethanol extract caused a greater tumor size reduction than feeding with curcumin (Yue et al., 2016a,b). The presence of turmerones increases curcumin accumulation inside colonocytes and could enhance curcumin antitumor activity in mice models. Bevacizumab is a monoclonal antibody targeting vascular endothelial growth factor. It has been used in combination with turmeric ethanol extract (including curcumin) for treatment of mice harboring HT-29 xenografts. Also, a combination therapy of turmeric extract plus bevacizumab treatment significantly inhibited tumor growth. These inhibitory effects were comparable with those of FOLFOX plus bevacizumab, with no observable side-effect induced by turmeric extract treatment while significant side effects were found in FOLFOX-treated mice (Table 3; Yue et al., 2016b). Potential synergistic effects of turmerones, curcumin, and bevacizumab could eventually allow a future reduction in this antibody dosage to patients, if applied in clinic. This would lead to reduction/prevention of some rare side effects associated to bevacizumab therapy, as thrombosis, arterial hypertension, proteinuria, perforation of the gastrointestinal tract, or nasal septum, wound healing abnormalities (which may lead to postoperative bleeding in CRC surgery), irreversible leuco-encephalopathy syndrome, allergic skin rash, and hypersensitivity reactions (including flashing, pruritus, arterial hypertension, rigors, broncho-constriction, chest pain, and sweats). These side effects also include rare spontaneous delayed (sometimes even several months after surgery) leakage from colon or rectal anastomosis after treatment with bevacizumab (Pavlidis and Pavlidis, 2013).

## Gossypol

Gossypol (Figure 2K) is a natural phenolic aldehyde derived from the cotton plant (*Gossypium*). Its antitumor properties have been studied in a variety of tumors since the 1980s, being currently evaluated in phase I and II clinical trials for its use as a single agent or in combination with other antitumor agents in a variety of hematologic, lymphoid, and solid tumors. Gossypol inhibits cell proliferation and induces apoptosis and autophagy in a variety of CRC cell lines. Also, it inhibits CRC growth in a mouse xenograft model after oral administration (Zhang et al., 2003; Lan et al., 2015). Gossypol sensitizes the antitumor activity of 5-FU, causing a synergistic cytotoxic effect in HT-29, HCT116, and RKO cells, compared with monotherapies (Table 3; Yang D. et al., 2015).

## Conclusions

As a general rule, designing of combinations involving a traditional chemotherapy drug (or radiotherapy protocol) plus one or more natural bioactive compounds (including in some cases well-known nutraceuticals), could be a promising approach in order to potentially achieve improvements in the partial or complete remission of CRC tumors; and at the same time this could minimize side effects which could be associated with this drug treatment or radiotherapy (neutropenia, diarrhea, cardiotoxicity, nephrotoxicity, hepatotoxicity, etc.) (Figure 3). Most synergistic effects of these combinations have been reported in *in vitro* and using animal tumor models and are due to antioxidant bioactivity, apoptosis induction (via the mitochondrial or extrinsic pathways) and/or cell cycle arrest (at any checkpoint).

These beneficial effects due to the addition of a natural bioactive to the canonical drug treatment, are enhanced by the fact that these natural compounds and nutraceuticals can reinforce the drug effective concentration, which is needed in order to achieve the same therapeutic result. Also, interestingly, in some cases, addition of the bioactive compound may allow to overcome the intrinsic or acquired chemo- or radio-resistance occurring in some tumor cells, as these plant or fungal compounds may modulate simultaneously diverse target pathways in the neoplastic cell, overcoming those altered cell regulatory routes which may be responsible for a particular resistance mechanism.

Finally, in many cases, these bioactives are small molecular weight compounds present in medicinal plants and foods, which would allow their potential easy oral administration, independently of painful or stressful administration methods (peritoneal, catheters, etc.). In the specific case of CRC therapy, this is a fact of enormous importance, as these molecules can easily reach the transformed colon mucosa cells.

## Author contributions

Introduction and resveratrol section were written by SR, flavonoids section was written by JF and IG, terpenoids section was written by CV and FL. Final revision was made by FL.

## Funding

Authors wish to thank to MINECO (Spanish Ministry or Economy and Competitiveness) for grant AGL-2010-20622.

### Conflict of interest statement

The authors declare that the research was conducted in the absence of any commercial or financial relationships that could be construed as a potential conflict of interest.
